# Supplementary material for: Ultrafast optical modulation of vibrational strong coupling in ReCl(CO)3(2,2-bipyridine)
Source: Nanophotonics. 2025 Nov 13;14(27):5437–48. doi: 10.1515/nanoph-2025-0471 (PMC12717908; doi:10.1515/nanoph-2025-0471)
Supplement: Supplementary file 1 — Supplementary Material Details [file j_nanoph-2025-0471_suppl_001.pdf]

SUPPLEMENTARY MATERIAL

Ultrafast optical modulation of vibrational strong coupling in $ReCl(CO)_3$(2,2-bipyridine)

Liying Chen,[1] Alexander M. McKillop,[1] Ashley P. Fidler,[1,2] and Marissa L. Weichman[1,*]

[1]Department of Chemistry, Princeton University, Princeton, New Jersey 08544, USA

[2]Present Address: Chemistry Division, Naval Research Laboratory, Washington, DC 20375, USA

[*]weichman@princeton.edu

Section S1: Temporal fitting of extracavity and intracavity vibrational dynamics

We perform time-domain fitting of the extracavity transient data for ReCl(CO)$_3$(2,2-bipyridine) discussed in Section 3.2 of the main text, as well as the intracavity $\Delta\alpha_t(\nu)$ datasets extracted using the spectral reconstruction algorithm detailed in Section 3.4. Temporal lineouts are taken at characteristic wavenumbers corresponding to the ground-state bleach (GSB, 2018 cm^{-1}) and the excited-state absorption (ESA, 2060 cm^{-1}). For the shifting ESA band, we additionally fit the feature with a Lorentzian function at each delay time in order to extract the center frequency as a function of time. The temporal evolution of the lineouts and frequency shifts are fit using exponentials to determine rise, decay, and shift time constants. Representative exponential fits are shown in Fig. S1 for the GSB rise dynamics, Fig. S2 for the GSB decay dynamics, Fig. S3 for the ESA decay dynamics, and Fig. S4 for the ESA shift. The left-hand panels of Figs. S1−S4 show fitting of the same representative extracavity dataset shown in the left-hand column of Fig. 4 in the main text. The right-hand panels of Figs. S1−S4 show fits for the same reconstructed intracavity dataset under resonant VSC shown in the right-hand column of Fig. 4. Extracted time constants averaged across all data sets are summarized in Table 1 of the main text.

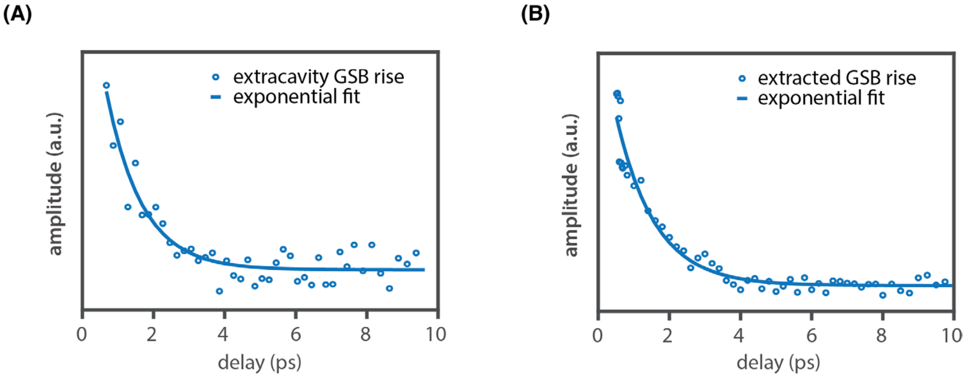

Figure S1 | Early time ground-state bleach (GSB) rise dynamics from lineouts taken at 2018 cm^{-1} in representative **(A)** extracavity data from the left-hand column of Fig. 4 and **(B)** intracavity data collected under resonant VSC and reconstructed using the algorithm detailed in Section 3.4 of the main text. Experimental data is plotted in points, while solid lines represent best-fit curves. To fit the early-time GSB rise, data points for $t < 10$ ps are fit with a single exponential function.

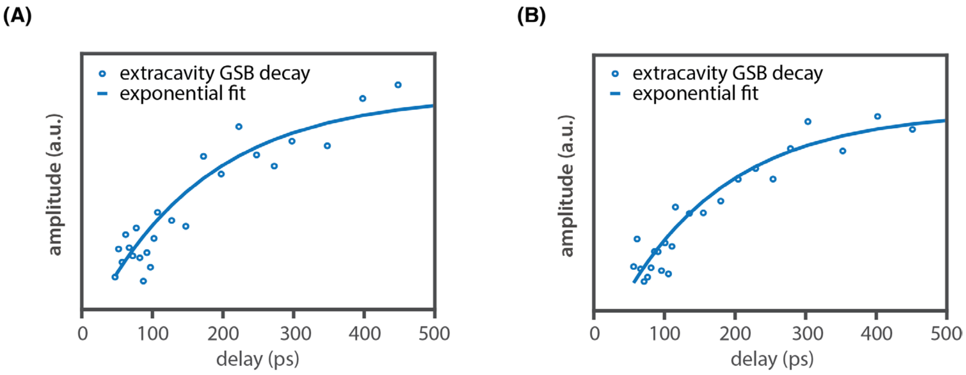

Figure S2 | Long-time ground-state bleach (GSB) decay dynamics from lineouts at 2018 cm^{-1} in representative **(A)** extracavity data from the left-hand column of Fig. 4 and **(B)** intracavity data collected under resonant VSC and reconstructed using the algorithm detailed in Section 3.4 of the main text. Experimental data is plotted in points, while solid lines represent best-fit curves. To fit the long-time GSB decay, data points for $t > 50$ ps are fit with a single exponential function.

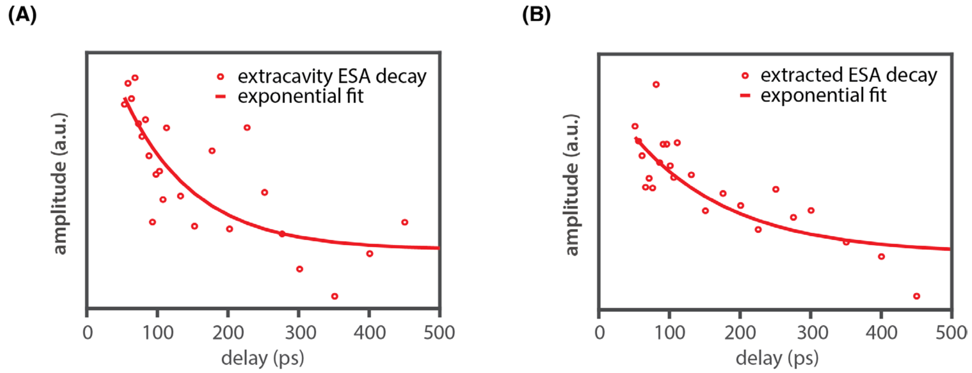

Figure S3 | Long-time excited state absorption (ESA) decay dynamics from lineouts at 2060 cm^{-1} in representative **(A)** extracavity data from the left-hand column of Fig. 4 and **(B)** intracavity data collected under resonant VSC and reconstructed using the algorithm detailed in Section 3.4 of the main text. Experimental data is plotted in points, while solid lines represent best-fit curves. To fit the long-time ESA decay, data points for $t > 50$ ps are fit with a single exponential function.

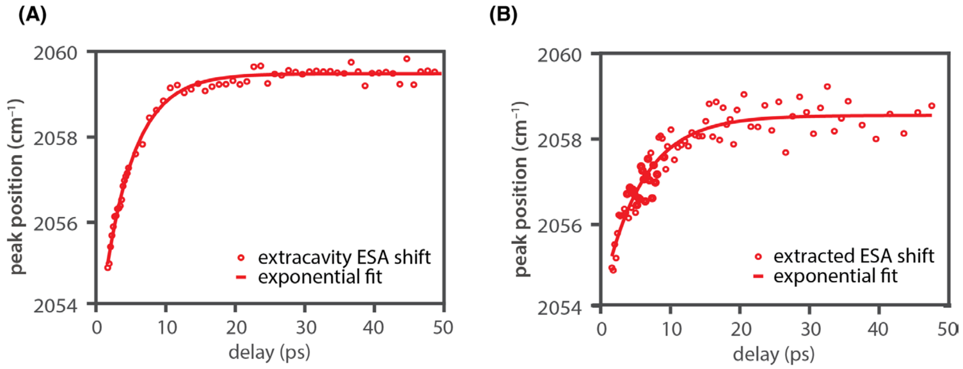

Figure S4 | Excited-state absorption (ESA) peak shift dynamics obtained from Lorentzian fitting of the ESA peak center as a function of pump-probe delay time in **(A)** extracavity data from the left-hand column of Fig. 4 and **(B)** intracavity data collected under resonant VSC and reconstructed using the algorithm detailed in Section 3.4 of the main text. Experimental data is plotted in points, while solid lines represent best-fit curves. Data points for $t < 50$ ps are fit with a single exponential function.

Section S2: Detuned cavity control experiments

To examine role of cavity detuning, we perform control experiments on intracavity $ReCl(CO)_3(bpy)$ in microfluidic FP cavities where the cavity length is adjusted away from exact resonance with the *a′* symmetric carbonyl stretch at 2018 cm^{-1}. We access these detuning conditions by laterally translating the cavity in the plane of the mirrors, taking advantage of slight deviations from perfect mirror parallelism to vary the effective cavity length at the location where the pump-probe measurement is performed.

Data from a representative UV-pump/IR-probe experiment performed for intracavity $ReCl(CO)_3(bpy)$ under detuned conditions are shown in Fig. S5. The left-hand column of Fig. S5 reproduces the same extracavity transient data as the left-hand column of Fig. 4 of the main text. The central column of Fig. S5 shows the raw transient cavity transmission data for the detuned device. Under these conditions, the static pump-off cavity transmission spectrum (Fig. S5B) exhibits two polaritonic peaks with unequal amplitudes and a splitting different from the resonant VSC case. The raw transient transmission spectra for the detuned cavity (Fig. S5EH) display derivative-like lineshapes rather than simple bleach or absorption features. The intracavity molecular response extracted with our spectral reconstruction algorithm is plotted in the right-hand column of Fig. S5. The reconstructed spectra clearly resolve the underlying molecular response, yielding clear GSB and ESA features. The extracted time constants for the temporal evolution of these features under detuned cavity-coupling conditions are summarized in Table 1 of the main text and are consistent with the extracavity control.

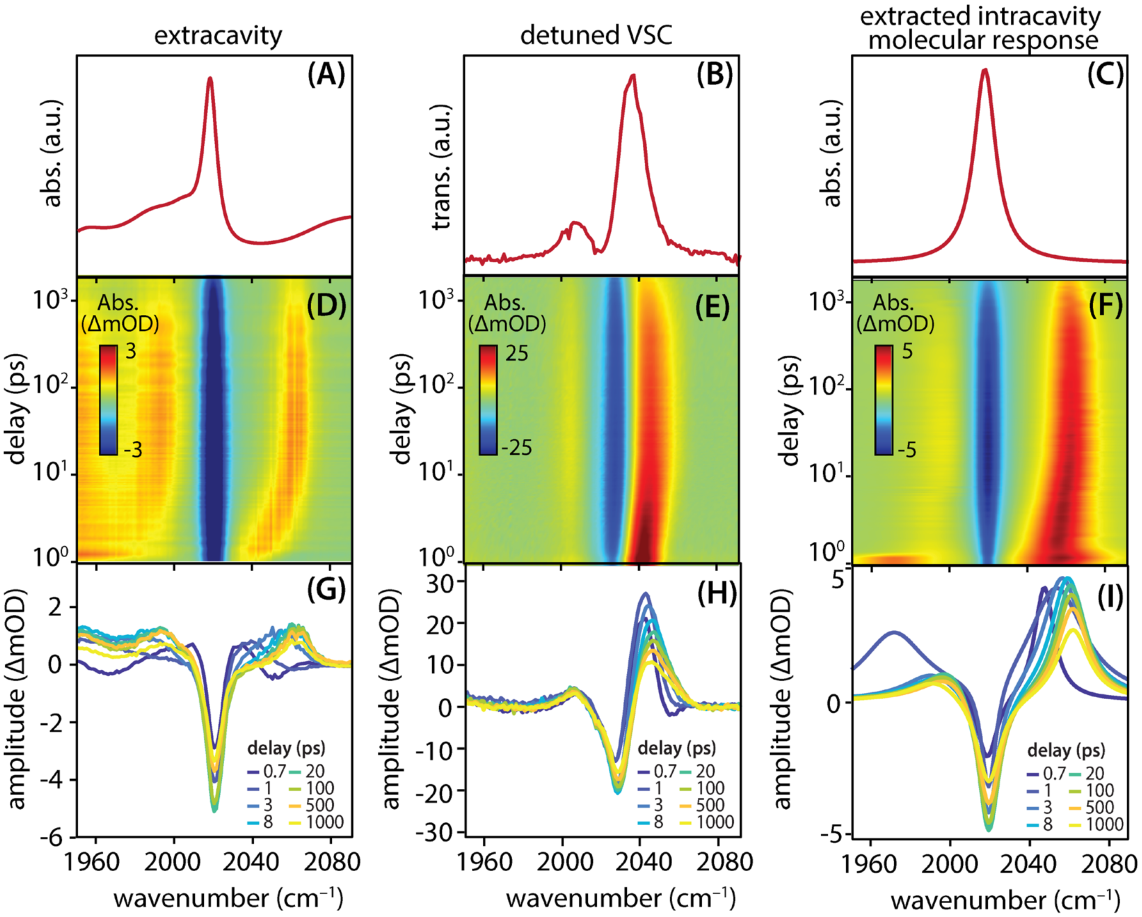

Figure S5 | Ultrafast UV-pump/IR-probe spectroscopy of $ReCl(CO)_3(bpy)$ in DMSO. Extracavity control data is presented in the left-hand column (reproduced from Fig. 4) and intracavity data collected under detuned VSC conditions is presented in the central column. The right-hand column presents the molecular response extracted from intracavity data, which can be compared directly to extracavity data. **(A)** Steady-state extracavity IR absorption spectrum of $ReCl(CO)_3(bpy)$ in DMSO showing the a' symmetric carbonyl stretching mode near 2018 cm^{-1}. **(B)** Static (pump-off) transmission spectrum of a detuned cavity filled with $ReCl(CO)_3(bpy)$ in DMSO, exhibiting asymmetric polariton features. **(C)** Representative pump-off intracavity absorption spectrum for $ReCl(CO)_3(bpy)$, $\alpha_0(\nu)$, modeled with a single Lorentzian line-shape and fit to the pump-off cavity transmission spectrum in the first step of the spectral reconstruction algorithm. **(D,E,F)** Ultrafast UV-pump/IR-probe spectra plotted as a function of delay time and wavenumber showing **(D)** the differential absorption of an extracavity sample, **(E)** the differential cavity transmission of an intracavity sample under detuned coupling conditions, and **(F)** the intracavity molecular response, $\Delta\alpha_t(\nu)$, reconstructed from the data in panel (E). **(G, H, I)** Spectral lineouts at selected pump-probe delays of transient data acquired for **(G)** the extracavity sample, **(H)** the intracavity sample, and **(I)** the reconstructed intracavity molecular response.

Section S3: Forward-construction of transient cavity transmission spectra

In the main text, we emphasize reconstruction of the intracavity transient absorption from the transient cavity transmission spectra, which can then be directly compared to extracavity control data. One can also "forward-construct" the transient cavity transmission spectrum by applying the Fabry-Pérot expression in Eq. (1) of the main text to extracavity transient absorption data. The constructed cavity transmission spectra can then be compared against raw cavity data. Figure S6 provides an example of how we also find good agreement between extracavity and intracavity spectra using this approach. Note, however, that interpretation of transient cavity spectra are more nuanced than transient absorption spectra, as discussed in Section 3.3 of the main text, making it more involved to interpret the features of these data than it is to understand the reconstructed intracavity molecular response data (Fig. 4).

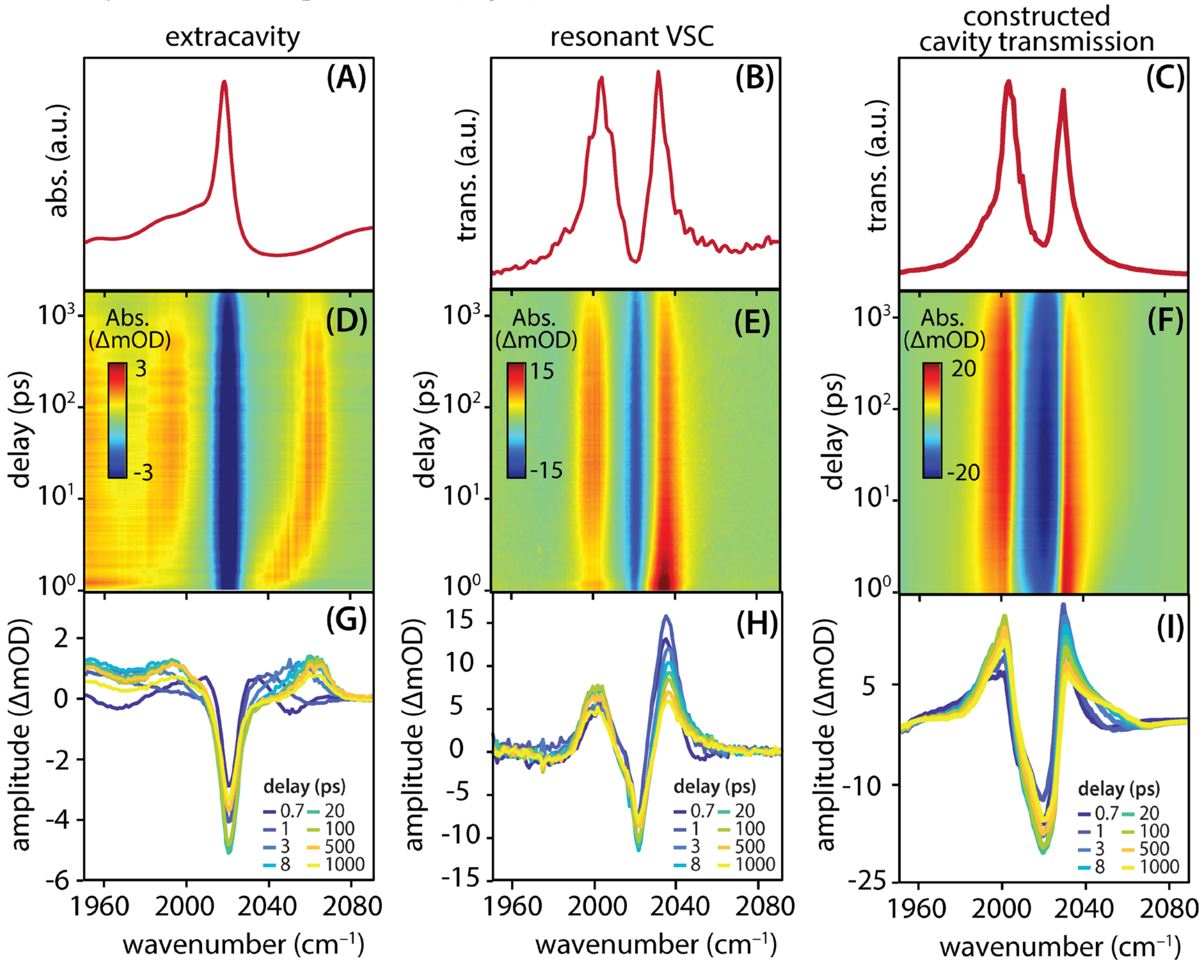

Figure S6 | Ultrafast UV-pump/IR-probe spectroscopy of $ReCl(CO)_3(bpy)$ in DMSO. **(A, D, G)** Extracavity data and **(B, E, H)** resonant intracavity data reproduced from Fig. 4. **(C, F, I)** Transient cavity transmission spectra constructed by processing the extracavity data in the left-hand column with Eq. (1) of the main text. **(C)** Simulated pump-off transmission spectrum of a 25.07 µm-long cavity with $R = 0.95$ mirrors, filled with dielectric material featuring an absorption coefficient given by the experimental extracavity data in panel (A). **(F)** Simulated differential UV-pump/IR-probe cavity transmission spectra plotted as a function of delay time and wavenumber. Transient traces are constructed by calculating pump-on and pump-off cavity transmission spectra from the corresponding pump-on and pump-off extracavity data that underlie the spectra in panel (D). **(I)** Spectral lineouts at selected pump-probe delays of transient data from panel (F).

Section S4: Reproducibility of transient extracavity and intracavity measurements

We collect multiple independent transient datasets for each condition (extracavity, intracavity under resonant VSC, and intracavity with detuned coupling) and extract time constants from each dataset using the fitting methods described in the main text and in Section S1. The results from independent datasets are summarized in Tables S1–S3. Table S1 compiles all time constants extracted from extracavity datasets, Table S2 presents results obtained under resonant VSC conditions, and Table S3 presents results obtained under detuned intracavity conditions. Averaged values and associated standard deviations for each dataset are reported in Table 1 of the main text.

For data processing, some time constants are discarded according to criteria designed to eliminate unreliable values. Any value falling more than three standard deviations from the mean for a given condition is considered an outlier and excluded. In some scans, particular kinetic components cannot be clearly resolved and are likewise discarded. Because we fit each kinetic component independently, exclusion of one component does not affect the inclusion of others in the same dataset. The remaining results demonstrate reproducibility across independent scans, with extracted time constants for extracavity and intracavity conditions all consistent within experimental uncertainty.

Table S1. Time constants obtained for the ground-state bleach (GSB) rise and decay and excited-state absorption (ESA) decay and shift from independent UV-pump/IR-probe experiments on extracavity $ReCl(CO)_3(bpy)$ in DMSO.

Extracavity dataset	GSB rise (ps)	GSB decay (ps)	ESA decay (ps)	ESA shift (ps)
2025-06-13-1	1.0	160	100	6.5
2025-06-13-4	1.5	150	120	6.2
2025-06-13-5	1.9	140	130	6.4
2025-06-13-8	1.6	150	140	6.5
2025-06-13-9	1.4	160	120	6.5
2025-06-13-10	1.6	140	170	6.5
2025-06-13-11	1.6	150	90	6.4
2025-06-13-12	1.5	140	140	6.8
2025-06-13-13	1.6	130	100	6.4
2024-06-24-3	0.9			6.1
2024-06-24-4	0.9			6.8
2024-06-24-5	1.4			5.8
2024-06-24-6	0.8			6.6
2024-06-05-2	1.2			
2024-06-05-4	1.4			4.5
2024-06-05-5	1.1			4.8
2024-06-06-1	1.0			
2024-06-11-2	1.7			6.7
2024-06-11-4	1.8			5.4
2024-06-11-5	1.2			5.4
2024-06-11-6	1.3			5.6
2024-06-11-7	1.5			6.5
2024-06-11-8	1.5			5.8
**Mean**	**1.4 ± 0.3**	**150 ± 10**	**120 ± 20**	**6.1 ± 0.7**

Table S2. Time constants obtained for the ground-state bleach (GSB) rise and decay and excited-state absorption (ESA) decay and shift from independent UV-pump/IR-probe experiments on intracavity $ReCl(CO)_3(bpy)$ in DMSO under resonant VSC of the *a′* symmetric carbonyl stretch at 2018 cm^{-1}.

Resonant VSC dataset	GSB rise (ps)	GSB decay (ps)	ESA decay (ps)	ESA shift (ps)
2025-06-20-1	1.0	160	160	4.2
2025-06-20-2	1.1	150	120	4.3
2025-06-20-3	1.7	180	190	4.6
2025-06-20-4	1.5	150	100	4.3
2025-06-20-5	1.3	200	170	5.1
2025-06-20-6	2.0	160	150	4.8
2025-06-20-7	1.2	150	140	3.0
2025-06-23-1	2.9	160	140	4.4
2024-07-02-1	1.5			
2024-07-02-2	1.4			
2024-07-02-3	1.5			
2024-07-02-4	1.2			7.2
2024-07-02-5	1.4			
2024-07-02-6	1.5			6.1
2024-07-02-9	1.2			6.5
2024-07-01-1	1.4			
2024-07-01-2	1.2			
2024-07-01-3	1.5			
2024-07-01-4	1.5			
2024-07-01-5	1.6			6.3
2024-06-27-1	1.9			6.2
2024-06-27-3	2.1			
2024-06-27-5	1.8			
2024-06-25-2	2.1			
**Mean**	**1.6 ± 0.4**	**170 ± 20**	**150 ± 30**	**5.2 ± 1.2**

Table S3. Time constants obtained for the ground-state bleach (GSB) rise and decay and excited-state absorption (ESA) decay and shift from independent UV-pump/IR-probe experiments on intracavity $ReCl(CO)_3(bpy)$ in DMSO under detuned intracavity conditions.

Detuned VSC dataset	GSB rise (ps)	GSB decay (ps)	ESA decay (ps)	ESA shift (ps)
2025-06-23-1	1.3	150	150	6.5
2025-06-23-2	1.7	160	140	6.5
2025-06-23-3	1.5	180	130	6.3
2025-06-24-1	2.0	130	140	4.0
2025-06-24-4		150	140	
2024-07-02-7	1.4	160	90	5.6
2024-06-25-6	1.7	110	100	7.1
**Mean**	**1.6 ± 0.3**	**150 ± 20**	**130 ± 30**	**6.0 ± 1.6**
